# Biologically-inspired neuronal adaptation improves learning in neural networks

**DOI:** 10.1080/19420889.2022.2163131

**Published:** 2023-01-17

**Authors:** Yoshimasa Kubo, Eric Chalmers, Artur Luczak

**Affiliations:** a Canadian Centre for Behavioural Neuroscience, University of Lethbridge, Lethbridge, AB, Canada; b Department of Mathematics & Computing, Mount Royal University, Calgary, AB, Canada

**Keywords:** Bio-plausible neural networks, neuronal adaptation, equilibrium propagation, contrastive Hebbian learning

## Abstract

Since humans still outperform artificial neural networks on many tasks, drawing inspiration from the brain may help to improve current machine learning algorithms. Contrastive Hebbian learning (CHL) and equilibrium propagation (EP) are biologically plausible algorithms that update weights using only local information (without explicitly calculating gradients) and still achieve performance comparable to conventional backpropagation. In this study, we augmented CHL and EP with *Adjusted Adaptation*, inspired by the adaptation effect observed in neurons, in which a neuron’s response to a given stimulus is adjusted after a short time. We add this adaptation feature to multilayer perceptrons and convolutional neural networks trained on MNIST and CIFAR-10. Surprisingly, adaptation improved the performance of these networks. We discuss the biological inspiration for this idea and investigate why Neuronal Adaptation could be an important brain mechanism to improve the stability and accuracy of learning.

## Introduction

Deep neural networks outperform humans in Atari games [1] and the Game of GO [2], but fall short of humans in tasks such as art, music, and translations. Thus, looking for inspiration from the brain may help to improve current deep neural networks. Many researchers are drawing inspiration from the brain to close the gap between biological and machine learning. For example, while the backpropagation algorithm (BP) has long been used to train deep neural networks by backpropagating error signals through the layers of the network [3], the debate over whether biological neurons could support such an operation [4,5] has led to several more biologically plausible algorithms being proposed [6–12]. This paper will focus on two of these: contrastive Hebbian learning (CHL) [13–15] and the closely related equilibrium propagation (EP) [12,16–18]. These algorithms model the network as a dynamical system and learn from temporal differences in activity rather than explicitly calculated errors or gradients.

CHL and EP consist of two learning phases: a free or negative phase, followed by a clamped or positive phase (note that these learning “phases” are not to be confused with the phases of sinusoidal components that make up the larger-scale brain rhythms, but are rather computational stages in CHL and EP models). During the free phase, an input signal is presented and the network is allowed to equilibrate to a steady state. The clamped phase then clamps output neurons to the desired targets, and the network is allowed to re-equilibrate. CHL clamps output neurons completely, while EP uses “soft” clamps, and nudges the output activities toward the desired level – which may be more biologically plausible. Weight updates are based on the differences between free- and clamped-phase activities of neurons on either side of the weight. Our previous study [19] showed that free-phase steady-state activity can be well predicted based on the first few steps of neural dynamics. This would mean that a full free-phase equilibration may not actually be required before output clamping, and that learning could occur without two distinct phases – making the EP concept even more biologically plausible.

In this study, we add *adjusted adaptation* to CHL and EP. This is based on the *Neural Adaptation* effect that has been observed in biological neurons in several systems [20,21]. During neural adaptation, a neuron’s response to a given stimulus will usually decrease over a short period of time from its initial level. This could be interpreted as a smooth change of sensitivity to the stimulus.

The neural adaptation phenomenon is well studied from both biological and computational perspectives. For example, the integrate-and-fire model of Hertäg et al. [22] included adaptation and reproduced realistic spiking behaviors. Jolivet et al. [23] showed that spike-frequency adaptation was a key feature for a computational model to accurately predict spike trains of real neurons. Reutimann et al. [24] studied the cortex of monkeys, suggesting that firing rate adaptation in inhibitory neurons causes climbing activity in the cortex which represents the passage of time and predicts the timing of important events. Stemmler et al. [25] derived an unsupervised learning rule describing how firing rates could be modulated for maximum information compression. Fontaine et al. [26] studied membrane potential variability in barn owls, concluding that it is a genuine feature of neurons as opposed to a result of noise. Carandini et al. [27] used a rectification model [28] to model the effect of adaptation in the cat visual cortex, and Treves [29] has discussed adaptation as a prerequisite for several key computational mechanisms throughout the brain [30–32]. Given the vast research on adaptation and its importance in the brain, here we suppose that adaptation may offer advantages for artificial neural networks and test its effect on networks trained to perform benchmark classification tasks.

Here, we model adaptation during the clamped phase, such that each neuron’s clamped-phase activity is gradually pushed back toward its free-phase activity. This reduces the difference between the free- and clamped-phase activations; modulating weight updates in a way that makes training smoother.

This paper extends our previous work [19,33] by applying the adaptation concept to multilayer perceptrons (MLPs) and convolutional neural networks (CNNs) trained on MNIST [34] and CIFAR-10 [35] by CHL and EP. We make the following contributions:

1. Demonstrate that models with adaptation achieve better performance than models without adaptation on both MNIST and CIFAR-10 tasks.
2. We provide an explanation of why adaptation may work by comparing the training gradients created with and without adaptation and comparing these to the gradients created by backpropagation.

## Method

In this section, we discuss some notations, methods, and model specifications for our experiments.

### EP and CHL

During the free phase of EP, the network calculates the dynamics of activity without any target signals or gradient signals. In a one-hidden-layer MLP, the equations of dynamics for activity at each layer are described based on previous work [12,16] as:

(1) $$x_{o,t}=x_{o,t-1}+h\left(-x_{o,t-1}+p\left(\underset{j}{\sum }w_{j,o}x_{j,t-1}+b_{o}\right)\right),$$

(2) $$x_{j,t}=x_{j,t-1}+h\left(-x_{j,t-1}+p\left(\overset{\;}{\underset{i}{\sum }}w_{i,j}x_{i,t-1}+\gamma \overset{\;}{\underset{o}{\sum }}w_{o,j}x_{o,t-1}+b_{j}\right)\right),$$

where *x* is an activity, *w* represents weights for each layer, *i, j*, and *o* are indexes of input, hidden, and output layer neurons, and *b* is a bias. *p* is an activation function such as the sigmoid function, and γ is the feedback parameter. *h* is the Euler method’s time-step. Please note that for consistency with our previous work [19], we use letter o for indexing output units. We hope that “o” will not be confused with number 0, which is not present in our equations.

During the clamped phase, the output neurons are influenced by target signals. During this phase, the network calculates the dynamics of activity in the output layer as:

(3) $$x_{o,t}=x_{o,t-1}+h\left(-x_{o,t-1}+p\left(\overset{\;}{\underset{j}{\sum }}w_{j,o}x_{j,t-1}+b_{o}\right)+\beta \left(y-x_{o,t-1}\right)\right),$$

where *y* is a target signal, and β is a nudging parameter that pushes output-layer activations back toward their free-phase level.

Given these activations at the free and clamped phases, weights will be updated by

(4) $$\Delta w_{pre,post}=\frac{1}{\beta }\alpha \left({\hat{x}}_{pre}{\hat{x}}_{post}-{\overset{\vee }{x}}_{pre}{\overset{\vee }{x}}_{post}\right),$$

where $\hat{x}$ is an activity at the clamped phase, $\overset{\vee }{x}$ is an activity at the free phase, α is the learning rate. *pre* and *post* are previous and post layer neuron indexes, respectively (e.g. for $\Delta w_{\left\{i,j\right\}}$, *pre* and *post* will be *i* and *j*, respectively).

CHL is very similar to EP. Instead of Equations 3 and 4, CHL uses:

(5) $$x_{o,t}=y$$

(6) $$\Delta w_{pre,post}=\alpha \left({\hat{x}}_{pre}{\hat{x}}_{post}-{\overset{\vee }{x}}_{pre}{\overset{\vee }{x}}_{post}\right)$$

Note that Equation 5 is used only for the clamped phase at the output layer. The free phase uses Equation 1 at the output layer. Thus, CHL clamps output neurons completely, where EP uses a “soft” clamping effect. The soft clamping weekly nudges the outputs at the free phase to their targets to minimize the difference between current outputs and targets. On the other hand, CHL uses a “hard” clamping, where the output neurons are clamped at the desired target value.

Note that when gaps between $\hat{x}$ and $\overset{\vee }{x}$ are large, especially, at the top layer, Δw would also be large – potentially leading to abrupt jumps in network weights. Softening the clamping by reducing β does not solve this problem, because the 1/β term in Equation 4 amplifies the differences. We can reduce α, but this slows learning convergence [36,37], and a smaller learning rate could lead the network to find sharp local minima [38].

### Predictive learning rule

In this subsection, we discuss our learning rule. Our rule modifies Equation 6 by replacing $\overset{\vee }{x}$_pre_ (the free-phase activity of the presynaptic neuron) with $\hat{x}$_pre_ (the *clamped-phase* activity of the presynaptic neuron). Our previous work [19] showed that a rule of this form emerges naturally if we assume that each neuron is working to maximize its metabolic energy. The new rule is as follows:

(7) $$\Delta w_{pre,post}\propto \frac{1}{\beta }\alpha \left({\hat{x}}_{pre}{\hat{x}}_{post}-{\hat{x}}_{pre}{\overset{\vee }{x}}_{post}\right)=\frac{1}{\beta }\alpha {\hat{x}}_{pre}\left({\hat{x}}_{post}-{\overset{\vee }{x}}_{post}\right)$$

**Figure 1.**
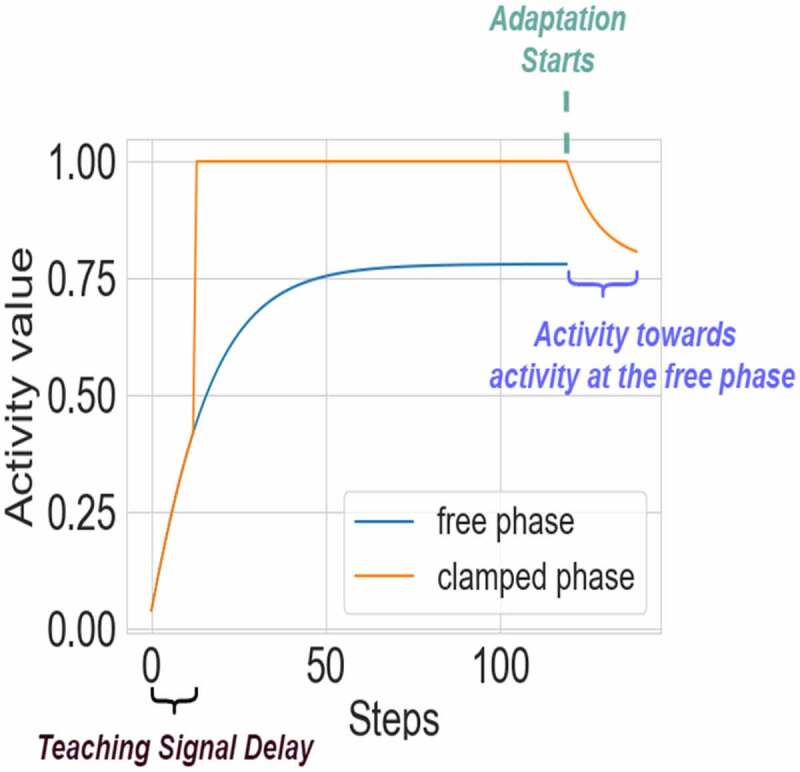
An example of neuron activity during the free and clamped phases with adjusted adaptation. For visual clarity, only one representative neuron from the top layer is shown. The time steps for free and clamped phases are 120, and teaching signals are given after 12 steps. The adjusted adaptation steps are 20. After 120 steps at the clamped phase, additional 20 steps for the adaptation are applied using Eq. 8.

In case of CHL, $\frac{1}{\beta }$ is removed. We call this update rule the *predictive learning rule* because our previous study used *predicted* free-phase steady-state activity ($\tilde{x}$) in place of $\overset{\vee }{x}$ (allowing neurons to predict their own free-phase steady state may be more biologically plausible, as it allows learning to occur without requiring two distinct phases). However, for the purpose of investigating adaptation, this study computes free-phase steady-state activities in the conventional way – which is the same as assuming perfect predictions of free-phase activity (a reasonable assumption, as our previous work found correlation between predicted ($\tilde{x}$) and actual free-phase activity ($\overset{\vee }{x}$) was *R* = 1 ± 0.0001 SD [19]). For consistency with this previous study [19], we apply delay to a clamped (teaching) signal to models trained by CHL. For example, Figure 1 shows that the signal is clamped after 12 steps. In our previous paper, we discuss that such delay could be more biologically plausible, as in the visual cortex, a top-down “teaching” signal (similar to clamped phase) arrives tens of microseconds later than the initial bottom-up signal (similar to the free phase).

### Adjusted adaptation

*Adjusted Adaptation* was introduced by our previous study [33], based on neural adaptation observed in the brain. To implement *Adjusted Adaptation*, the activities at the clamped phase are nudged toward activities at the free phase to reduce the gap between these activities. We speculate that these smaller gaps give smoother weight updates compared to conventional EP. We model adaptation as follows:

(8) $${\hat{x}}_{adp,t+1}=\left(1-c\right){\hat{x}}_{adp,t}+c*\overset{\vee }{\underset{*}{x}}$$

where *c* is a coefficient parameter, $\overset{\vee }{\underset{*}{x}}$ is a steady state at the free phase. Equation 8 is applied during additional steps after computing the clamped-phase dynamics. The form of Equation 8 was chosen to allow for a simple formula to vary the strength of adaptation. This formulation results in smooth, exponential change in neuronal activity across time steps (Figure 1, steps: 120–140), which is similar to changes observed experimentally in actual neurons during adaptation [20]. However, the exact relation of our parameter *c* to the biological time constant(s) of adaptation will be for future work as it may require conducting experimental work to properly relate the two.

We describe this algorithm as pseudocode next to Figure 1. Figure 1 depicts the adapted dynamics of activities during a CHL clamped phase. In this case, the clamped (teaching) signal is given 12 steps after the input signal is presented, as explained earlier. Adaptation is applied after 120 steps, and nudges the clamped phase activity back toward free-phase activity. The input signal is constant (not changing) throughout free and clamped phase. Details of the training process of our model with adaptation are shown in Figure 1 in Algorithm 1.

**Algorithm 1** Basic algorithm to train our models

**Figure:**
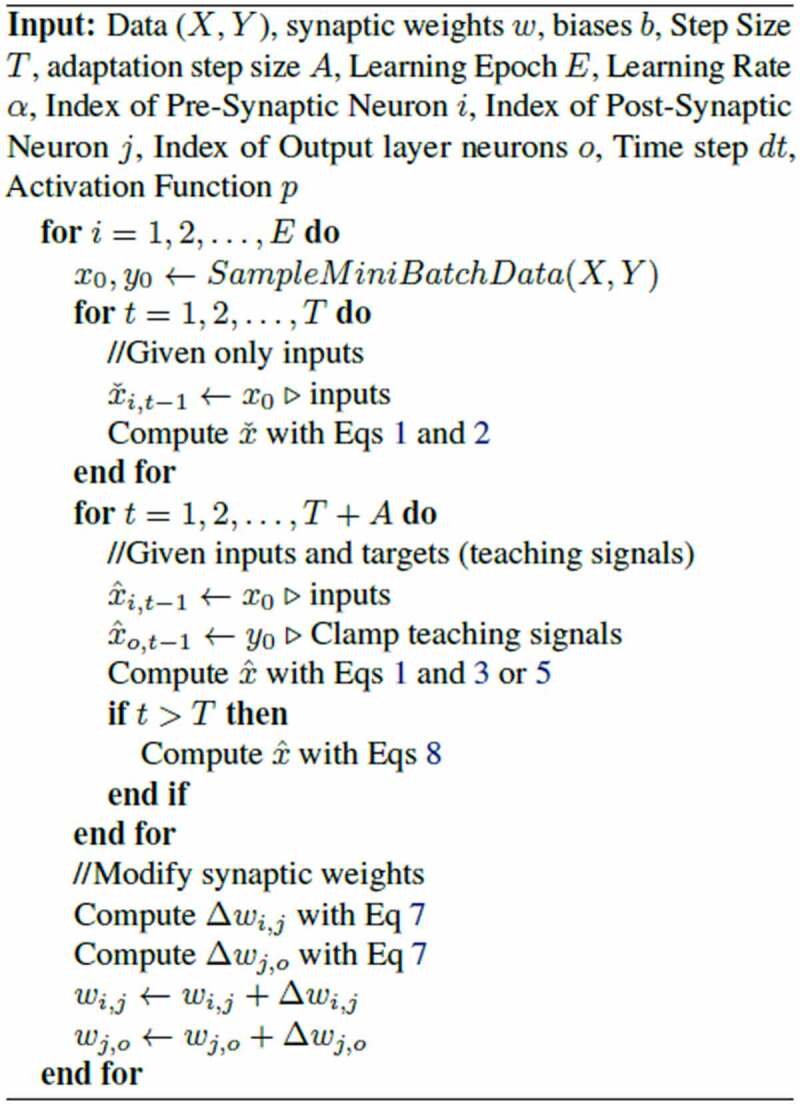

### Model specifications

For MLP with CHL on the MNIST dataset, the number of time steps for the free phase and clamped phases was set to 120. We tried seven different architectures: 782-6-10 (meaning the number of neurons at input–hidden–output layers), 782-50-10, 782-50-10 with lateral connection on the hidden layer, 782-50-10 with lateral connection on both the hidden and top layer, 782-1000-10, 782-1000-10 with lateral connection on the hidden layer, 782-1000-10 with lateral connection on both the hidden and top layer. We conducted these experiments with different architecture to check that we still get robust results on our models even if we change parameters. We used the learning rate of 0.1 on 782-6-10 and 782-50-10 models, and learning rate 0.03 for 782-1000-10 models. The teaching signal delay was 12 steps and *h* was 0.1 for all the networks. For our model with the adjusted adaptation, extra steps for adjusted adaptation are 20 and *c* was 0.1 (*c* = 0.1 gave the best performance in our simulations; however, a wide range values of *c* between 0.01 and 0.2 also resulted in consistent improvements over the model with no adaptation; we did not test extensively values of *c* above 0.2 because larger *c* diminishes impact of clamped (teaching) signal which leads to a very slow learning in the network). All models use the sigmoid activation function. For all experiments, we used the AdaGrad optimizer [39] (to find how to implement CHL with AdaGrad please see our code at https://github.com/ykubo82/bioCHL/blob/add-license-1/CHL_clamped.py, specifically Line 107).

For the CNNs with EP on the CIFAR-10 dataset, the time steps for the free phase and clamped phases are 130 and 30, respectively, and *β* is 0.18. We set the learning rates for the network to 0.21, 0.021, and 0.021 for the first, second convolutional layer, and fully connected layer, respectively. Our model consists of 256 and 512 filters whose sizes are 3 × 3 for both 1st and 2nd layers and followed by one dense layer for the output layer. *h* is 1.0 for this model. For the clamped phase, activities from the free phase at time step 110 are used as the initial activities (this is the same as teaching signal delay). This can be seen as a delay similar to our previous experiments. For our model with the adjusted adaptation, extra steps for implementing adaptation are 10 and *c* is 0.1. The activation function for these models is the hard sigmoid function [17]. We did not use any optimizer for these CNN models. Our CNN models are based on [16] and [33].

Code for our networks showing all the implementation details is available at https://github.com/ykubo82/AdpNet

## Results

**Figure 2.**
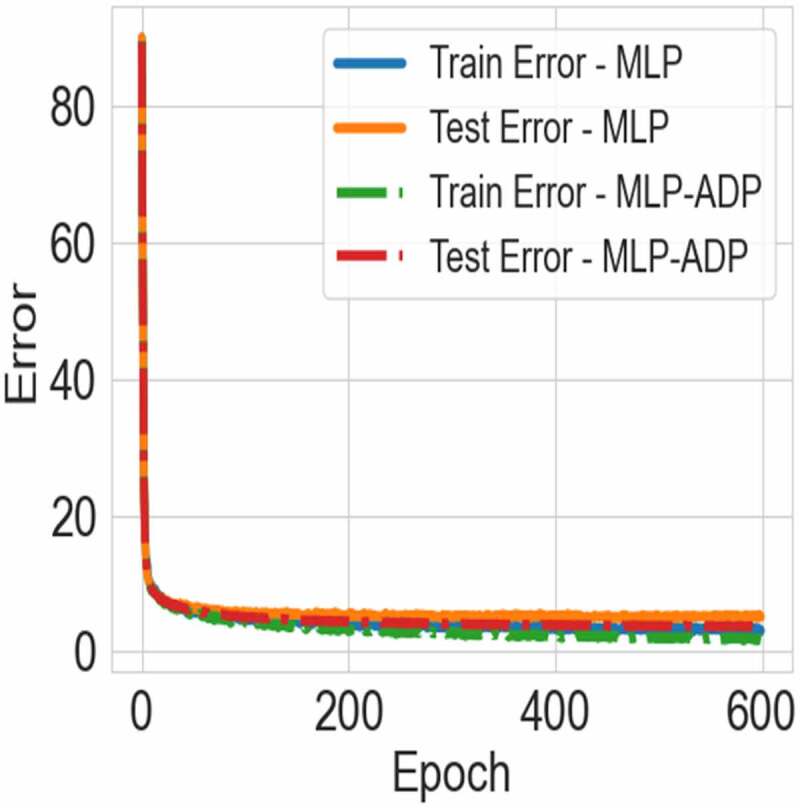
The learning curves for the models (parameters: 782-50-10) with the adjusted adaptation (MLP-ADP) and without the adjusted adaptation (MLP) on MNIST.

**Figure 3.**
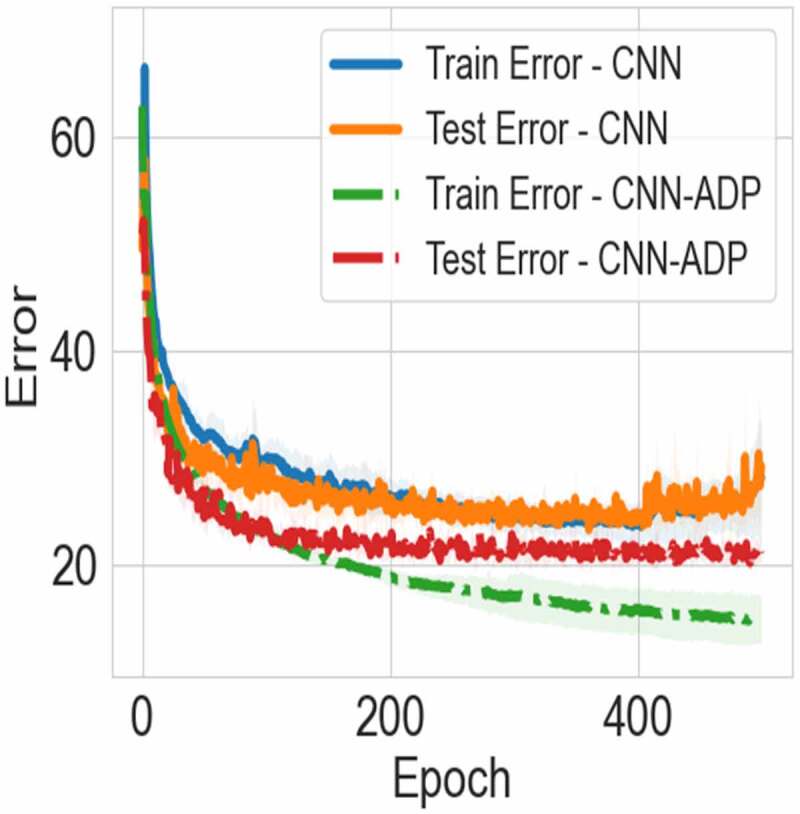
The learning curves for the models with the adjusted adaptation (CNN-ADP) and without the adjusted adaptation (CNN) on CIFAR-10.

Table 1 and Figure 2 show the results for MLP with/without the adaptation on the MNIST dataset. We found that adaptation improved performance, for all tested models. Similarly, CNNs with the adjusted adaptation (CNN-ADP) on CIFAR-10 achieved a better test error of 19.51 ± 0.6% as compared to the model without the adjusted adaptation (CNN, a test error of 22.46 ± 0.59%). Figure 3 shows the learning curves for these models. Note that the learning curves for models with adaptation are smoother. We consider this smoothness to be the result of adaptation, which reduces the gap between activities at the free and clamped phase.

**Table 1.** Training results on MNIST and CIFAR-10 with EP and the adaptation/no adaptation. For MNIST, we trained MLP with CHL. For CIFAR-10, we trained CNNs with EP. For *laterl1*, the lateral connections are only in the hidden layer. For *lateral2*, the lateral connections are in both hidden and output layers. We trained each network six times to calculate average and ±the standard deviation. Numbers in bold denote smallest test error.

	No Adaptation (error %)		Adaptation (error %)	
	Test	Train	Test	Train
MNIST (MLP)				
784-6-10	17.29 ± 1.78	16.11 ± 1.77	**11.93 ± 0.84**	9.45 ± 1.06
784-50-10	4.63 ± 0.19	2.71 ± 0.22	**3.56 ± 0.1**	1.62 ± 0.09
784-50-10; lateral 1	4.56 ± 0.30	2.54 ± 0.24	**3.82 ± 0.13**	1.76 ± 0.12
784-50-10; lateral 2	4.88 ± 0.33	2.81 ± 0.36	**3.86 ± 0.18**	1.84 ± 0.11
784-1000-10	1.98 ± 0.04	0.00 ± 0.00	**1.77 ± 0.06**	0.01 ± 0.00
784-1000-10; lateral 1	1.85 ± 0.05	0.00 ± 0.00	**1.78 ± 0.06**	0.01 ± 0.00
784-1000-10; lateral 2	1.83 ± 0.06	0.00 ± 0.00	**1.81 ± 0.07**	0.01 ± 0.00
CIFAR10 (CNN)				
256-512	22.46 ± 0.59	22.88 ± 1.71	**19.51 ± 0.6**	14.70 ± 2.31

In previous work, we already showed that our model even without adaptation achieved error rate similar to neural networks with comparable architecture trained with the BP algorithm on MNIST dataset [19]. Moreover, previously, we directly compared our model with the BP algorithm in a convolutional network trained on the CIFAR-10 dataset. To ensure the generality of that comparison, we repeated the training with BP three times using different learning rates for each simulation. Using BP, we achieved the smallest error rate of 21.23%, which was similar to our algorithm when applied to the same convolutional network (error rate of 20.88%) [19]. Therefore, here we did not run the same comparisons to BP, as it was already published before.

### Gradient checks

**Figure 4.**
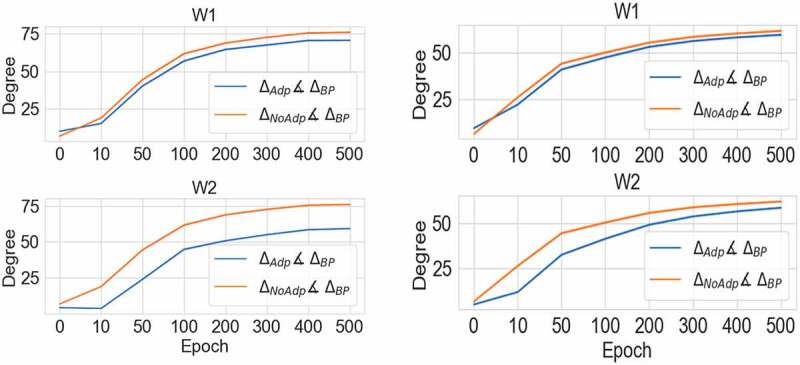
Mean angle between weight gradients calculated using BP and CHL (left: 6 neurons a hidden layer, right: 12 neurons in a hidden layer). Blue line shows the angle calculated for CHL with adaptation, and orange for CHL without adaptation. “W1” means the gradients for the weights between the input and hidden layers, and “W2” means the gradients for the weights between the hidden and output layers. The angle between the gradients with the adaptation and BP is denoted as ∆Adp∡∆BP . The angle between the gradients without the adaptation and BP is denoted as ∆NoAdp∡∆BP . Note that the blue line is consistently closer to 0, which demonstrates that CHL with adaptation provides weights updates more similar to BP gradients.

Why does adaptation work? To answer this question, we calculated the angles for the weights’ gradients between CHL models with/without the adaptation and models trained with backpropagation on the MNIST dataset. These gradients were calculated without applying the optimizer. This comparison is inspired by Lillicrap et al. [40]. Specifically, we calculated the angle θ between two weight gradient vectors, *a* and *b*:

(9) $$\theta =\cos^{-1}(\frac{||a\cdot b||}{||a||\cdot ||b||})$$

where each value in vector *a* represents a single weight update calculated as explained above: $\Delta w_{\left\{pre,post\right\}}=\alpha {\hat{x}}_{pre}\left({\hat{x}}_{post}-{\overset{\vee }{x}}_{post}\right)$. Similarly, corresponding values in vector *b* represent change in weights calculated using BP method, in the same network. While the gradients for BP are not always optimal in general (sometimes BP converges to local minima), BP is currently the gold standard for neural network training, and thus a good comparator. We found that weights’ gradients calculated in a network with adaptation were closer to BP gradients, than gradients without adaptation (Figure 4). Note that while the shape of the curves in Figure 4 is similar (indicating that the learning process progresses at similar rates), however, the curve for adaptation (blue) is always closer to 0°, indicating that adaptation consistently produces individual updates that are closer to BPs. Moreover, to ensure a fair comparison between gradients for the networks with and without the adaptation, we used the same initial weights to calculate the gradients for the networks with and without the adaptation. This might be one of the reasons for the similar shape of the curves. In addition, we also confirmed the robustness of our results in a network with a larger number of neurons on MNIST dataset (Figure 4 right side). In summary, our results suggest that adaptation changes network dynamics in a way that makes weight gradients closer to BP.

The meaning of these angles is best understood as the difference in the direction that each network is moving through the multidimensional parameter space during training. An angle of zero would mean, for example, that every weight that is increasing/decreasing in one network is also increasing/decreasing in the other. The fact that the networks with adaptation have lower angles in Figure 4 shows that adaptation makes weight updates more similar to those that would be experienced under BP.

For sanity check, we also tested that implementing adaptation is not simply equivalent to reducing a learning rate. To test it, we trained MLP (782-6-10) without the adaptation but with learning rates reduced to (1) 0.05, (2) 0.01, and (3) 0.001. We trained these models until the test error started increasing. The smallest test errors for networks with the learning rates (1), (2), and (3) were 16.03 ± 1.38%, 13.95 ± 0.39%, and 13.22 ± 1.25%, respectively. This shows that networks without adaptation and with smaller learning rates still have larger test errors as compared to the network with adaptation (11.93 ± 0.84%; learning rate: 0.1).

In addition, we also checked angles in the network on the CIFAR10 dataset. The mean angle between weight gradients calculated using BP and our model with adaptation was ∆Adp∡∆BP = 8.1° ± 4.29 SD. The model with no adaptation resulted in angles larger on average by 1.06° ± 0.3SD. Thus, analyses of networks on CIFAR10 and MNIST datasets provide consistent results.

## Conclusions

Neural adaptation has been observed in all types of neurons in vertebrates, as well as in invertebrates [20,21]. Neuronal adaptation can be defined as a change in activity over time in response to the same sensory stimulus, like sound, light, or tactile stimulation. Usually, neuron activity adapts most rapidly at the beginning, and plateauing at a steady-state value; similarly as implemented in our model (Figure 1). Interestingly, it was also proposed that neuronal adaptation could be a brain mechanism for surprise minimization, which may underlie conscious perception [33]. Considering that neural adaptation is a ubiquitous phenomenon across neuronal systems, thus, it may serve an important function in neural information processing. Here, we provide the first quantitative account of how neuronal adaptation can improve learning in deep neural networks.

Such improved learning could be due to the fact that if activity in the clamped phase is much different from activity without clamp, then learning may deteriorate as those two network states could be in different modes of the energy function [10,12,33]. Adaptation may thus reduce this problem by bringing clamped state closer to already learned state (free phase). This could also have a more cognitive interpretation. For that, let us use an analogy: if part of a car is occluded by a tree, then purely on sensory information, we cannot say what is behind that tree. However, based on what we learned about the world so far, we know what shape has a car, and thus we can assume that the rest of the car is likely behind the tree. Similarly, neuronal adaptation may allow a network to use already learned information (manifested by activity in free phase) as a strong context to more appropriately store new information (clamped signal).

We also note that adaptation could provide regularization effect: if there is a large difference between a particular neuron’s clamped and free-phase activities, that neuron would experience a stronger push back toward free-phase activity. The result would be a reduction of that neuron’s effect on the overall network’s direction during learning. Thus, adaptation could be seen as a new activation regularization method, similar to the commonly used dropout method [41], which is also an activation regularization.

It is also interesting to ask why an adaptation would make weight updates more similar to those calculated with BP. The most likely explanation for those results could be provided based on earlier theoretical work, which showed equivalence between CHL and BP [10,42], and equivalence of EP and BP [17,18], under certain assumptions. Despite different assumptions in those theoretical derivations (e.g. different network architectures), the common result is that when difference between activity in clamped and free phase approaches zero, then the gradients become proportional to error derivatives calculated with BP. In other words, if the change in network state caused by clamping the output neurons is infinitesimal, then it is equivalent to the error signal spread by backpropagation, except for a scalar prefactor [42]. In our model, an adaptation reduces exactly that difference between activity in clamped and free phase for each neuron. Therefore, an adaptation could result in gradients which follow more closely BP gradients. This suggests an intriguing idea that biology may use adaptation to implement a better approximation of the BP algorithm, which in many practical applications is a very effective learning algorithm. However, the meaning of the angles being quite large (e.g. >50°) is not entirely clear to us. It is possible that there are many ways to solve a given classification problem, and that EP tends to learn a different solution than BP (but adaptation makes the solution more like that found by BP).

It is also interesting to notice that our model is consistent with oscillatory brain activity. We suggest that a single ~2–20 Hz oscillation in the brain, consisting of ~50–500 ms long burst of neuronal population activity, can perform computation as in our algorithm. For example, the initial steps 1–12 in Figure 1 could correspond to activity evoked by the bottom-up sensory information. The following clamped phase in steps 13–120 may correspond to activity driven by top-down feedback information from higher-order areas. This activity is then modified toward levels expected from the initial bottom-up signals, which corresponds to adaptation in steps 121–140 in Figure 1. All those “sub-stages” may be seen as nested oscillations within a single slower oscillation which encompasses steps 1–140 in Figure 1. Interestingly, this idea implies that sensory information may be processed in discrete units, and each <~20 Hz oscillation may represent computation of a single percept [19]. For instance, this could explain why we cannot process visual information faster than ~20 frames per second, as it can take ~50 ms for the brain to evaluate if a single image is consistent with expectation, and only after that the next image could be processed in the next oscillation. However, more experimental work is needed to investigate how our model could be related to the nested network of rhythms or clocks in the brain.

In future work, we plan to apply models with adaptation to neuronal data analyses [43–47] and to reinforcement learning tasks [48,49]. Regularization effect of adaptation may help to improve training networks with BP. This might also help us to better understand the biological function of adaptation.

## References

[1] Mnih V, Kavukcuoglu K, Silver D, et al. Human-level control through deep reinforcement learning. Nature. 2015;518(7540):529–9. 25719670 10.1038/nature14236

[2] Silver D, Huang A, Maddison CJ, et al. Mastering the Game of Go with deep neural networks and tree search. Nature. 2016;529(7587):484–489. 26819042 10.1038/nature16961

[3] Rumelhart DE, Hinton GE, Williams RJ. Learning internal representations by error propagation. La Jolla, CA: California Univ San Diego La Jolla Inst for Cognitive Science; 1985.

[4] Crick F. The recent excitement about neural networks. Nature. 1989;337(6203):129–132. 2911347 10.1038/337129a0

[5] Lillicrap TP, Santoro A, Marris L, et al. Backpropagation and the brain. Nat Rev Neurosci. 2020;21(6):335–346. 32303713 10.1038/s41583-020-0277-3

[6] Bengio Y. How auto-encoders could provide credit assignment in deep networks via target propagation. arXiv preprint arXiv. 2014:1407.7906.

[7] Hinton GE, McClelland J. In Proceedings of the 1987 International Conference on Neural information processing systems; 1987 Jan 1. Learning representations by recirculation; p. 358–366.

[8] Lecun Y. Modeles connexionnistes de l’apprentissage (connectionist learning models) [PhD thesis]; 1987.

[9] Lillicrap TP, Cownden D, Tweed DB, et al. Random synaptic feedback weights support error backpropagation for deep learning. Nat Commun. 2016;7(1):1–10. 10.1038/ncomms13276 PMC5105169 27824044

[10] Movellan JR. Contrastive Hebbian learning in the continuous Hopfield model. In: Morgan Kaufmann, editor. Connectionist models. San Francisco, CA: Elsevier; 1991 Jan 1. p. 10–17.

[11] O’Reilly RC. Biologically plausible error-driven learning using local activation differences: the generalized recirculation algorithm. Neural Comput. 1996;8(5):895–938.

[12] Scellier B, Bengio Y. Equilibrium propagation: bridging the gap between energy-based models and backpropagation. Front Comput Neurosci. 2017;11:24. 28522969 10.3389/fncom.2017.00024 PMC5415673

[13] Almeida LB. A learning rule for asynchronous perceptrons with feedback in a combinatorial environment. In: Caudil M and Butler C, Editors. Proceedings of the IEEE First International Conference on Neural Networks San Diego, CA; 1987. p. 609–618.

[14] Baldi P, Pineda F. Contrastive learning and neural oscillations. Neural Comput. 1991;3(4):526–545. 31167332 10.1162/neco.1991.3.4.526

[15] Pineda FJ. Generalization of back-propagation to recurrent neural networks. Phys Rev Lett. 1987;59(19):2229. 10035458 10.1103/PhysRevLett.59.2229

[16] Ernoult M, Grollier J, Querlioz D, Bengio Y, Scellier B. Updates of equilibrium prop match gradients of backprop through time in an RNN with static input. Adv Neural Inf Process Syst. 2019;32.

[17] Laborieux A, Ernoult M, Scellier B, et al. Scaling equilibrium propagation to deep convnets by drastically reducing its gradient estimator bias. Front Neurosci. 2021;15:129. 10.3389/fnins.2021.633674 PMC7930909 33679315

[18] Scellier B, Bengio Y. Equivalence of equilibrium propagation and recurrent backpropagation. Neural Comput. 2019;31(2):312–329. 30576611 10.1162/neco_a_01160

[19] Luczak A, McNaughton BL, Kubo Y. Neurons learn by predicting future activity. Nature Mach Intell. 2022;4(1):1–11. 10.1038/s42256-021-00430-y PMC9262088 35814496

[20] Benda J. Neural adaptation. Curr Biol. 2021;31(3):R110–R116. 33561404 10.1016/j.cub.2020.11.054

[21] Whitmire CJ, Stanley GB. Rapid sensory adaptation redux: a circuit perspective. Neuron. 2016;92(2):298–315. 27764664 10.1016/j.neuron.2016.09.046 PMC5076890

[22] Hertã¤g L, Durstewitz D, Brunel N. Analytical approximations of the firing rate of an adaptive exponential integrate-and-fire neuron in the presence of synaptic noise. Front Comput Neurosci. 2014;8:116. 25278872 10.3389/fncom.2014.00116 PMC4167001

[23] Jolivet R, Rauch A, Lüscher HR, Gerstner W. Integrate-and-fire models with adaptation are good enough. Adv Neural Inf Process Syst. 2005;18:595–602.

[24] Reutimann J, Yakovlev V, Fusi S, Senn W. Climbing neuronal activity as an event-based cortical representation of time. J Neurosci. 2004;24(13):3295–3303. 15056709 10.1523/JNEUROSCI.4098-03.2004 PMC6730018

[25] Stemmler M, Koch C. How voltage-dependent conductances can adapt to maximize the information encoded by neuronal firing rate. Nat Neurosci. 1999;2(6):521–527. 10448216 10.1038/9173

[26] Fontaine B, Peña JL, Brette R. Spike-threshold adaptation predicted by membrane potential dynamics in vivo. PLoS Comput Biol. 2014;10(4):e1003560. 24722397 10.1371/journal.pcbi.1003560 PMC3983065

[27] Carandini M, Ferster D. Membrane potential and firing rate in cat primary visual cortex. J Neurosci. 2000;20(1):470–484. 10627623 10.1523/JNEUROSCI.20-01-00470.2000 PMC6774139

[28] Granit R, Kernell D, Shortess G. Quantitative aspects of repetitive firing of mammalian motoneurones, caused by injected currents. J Physiol. 1963;168(4):911. 14072865 10.1113/jphysiol.1963.sp007230 PMC1359475

[29] Treves A. Learning to predict through adaptation. Neuroinformatics. 2004;2(3):361–365. 15365197 10.1385/NI:2:3:361

[30] Treves A. Computational constraints that may have favoured the lamination of sensory cortex. J Comput Neurosci. 2003;14(3):271–282. 12766428 10.1023/a:1023213010875

[31] Treves A. Computational constraints between retrieving the past and predicting the future, and the CA3‐CA1 differentiation. Hippocampus. 2004;14(5):539–556. 15301433 10.1002/hipo.10187

[32] Treves A. Frontal latching networks: a possible neural basis for infinite recursion. Cogn Neuropsychol. 2005;22(3–4):276–291. 21038250 10.1080/02643290442000329

[33] Luczak A, Kubo Y. Predictive neuronal adaptation as a basis for consciousness. Front Syst Neurosci. 2021;15. DOI:10.3389/fnsys.2021.767461 PMC8789243 35087383

[34] LeCun Y, Bottou L, Bengio Y, et al. Gradient-based learning applied to document recognition. Proc IEEE. 1998;86(11):2278–2324.

[35] Krizhevsky A, Hinton G. Learning multiple layers of features from tiny images; 2009.

[36] Li F-F. CS231n: convolutional neural networks for visual recognition; 2021 [cited 2021 October 1]. Available from: https://cs231n.github.io/neural-networks-3/#annealing-the-learning-rate

[37] Sun J, Niu Z, Innanen KA, Li J, Trad D. A deep learning perspective of the forward and inverse problems in exploration geophysics. CSEG Geoconvention; 2019.

[38] Li Y, Wei C, Ma T. Towards explaining the regularization effect of initial large learning rate in training neural networks. Adv Neural Inf Process Syst. 2019;33:32.

[39] Duchi J, Hazan E, Singer Y. Adaptive subgradient methods for online learning and stochastic optimization. J Mach Learn Res. 2011;12(7):2121–2159.

[40] Lillicrap TP, Cownden D, Tweed DB, Akerman CJ. Random synaptic feedback weights support error backpropagation for deep learning. Nature communications. 2016;7(1): 1–10. 10.1038/ncomms13276 PMC5105169 27824044

[41] Srivastava N, Hinton G, Krizhevsky A, Sutskever I, Salakhutdinov R. Dropout: a simple way to prevent neural networks from overfitting. J Mach Learn Res. 2014;15(1):1929–1958.

[42] Xie X, Seung HS. Equivalence of backpropagation and contrastive Hebbian learning in a layered network. Neural Comput. 2003;15(2):441–454. 12590814 10.1162/089976603762552988

[43] Luczak A, Hackett TA, Kajikawa Y, et al. Multivariate receptive field mapping in marmoset auditory cortex. J Neurosci Methods. 2004;136(1):77–85. 15126048 10.1016/j.jneumeth.2003.12.019

[44] Luczak A, Narayanan NS. Spectral representation—analyzing single-unit activity in extracellularly recorded neuronal data without spike sorting. J Neurosci Methods. 2005;144(1):53–61. 15848239 10.1016/j.jneumeth.2004.10.009

[45] Ponjavic-Conte KD, et al. Neural correlates of auditory distraction revealed in theta-band EEG. Neuroreport: 2012;23(4):240–245. 22314684 10.1097/WNR.0b013e3283505ac6

[46] Ryait H, Bermudez-Contreras E, Harvey M, et al. Data-driven analyses of motor impairments in animal models of neurological disorders. PLoS Biol. 2019;17(11):e3000516. 31751328 10.1371/journal.pbio.3000516 PMC6871764

[47] Schjetnan AGP, Luczak A. Recording large-scale neuronal ensembles with silicon probes in the anesthetized rat. J Vis Exp. 2011;56:e3282. 10.3791/3282 PMC3227202 22042361

[48] Chalmers E, Luczak A. Reinforcement learning with brain-inspired modulation can improve adaptation to environmental changes. arXiv preprint arXiv. 2022;2205.09729.

[49] Kubo Y, Chalmers E, Luczak A. Combining backpropagation with equilibrium propagation to improve an actor-critic reinforcement learning framework. Front Comput Neurosci. 2022;16:980613. 36082305 10.3389/fncom.2022.980613 PMC9446087

